# Spontaneous esophageal rupture managed with endoscopic closure using an over-the-scope clip: A case report

**DOI:** 10.1016/j.ijscr.2021.105691

**Published:** 2021-02-22

**Authors:** Hirokatsu Hayashi, Narutoshi Nagao, Kenji Yamazaki, Ryuichi Asai, Chihiro Tanaka, Masahiko Kawai

**Affiliations:** a Department of Surgery, Gifu Prefectural General Medical Center, 4-6-1 Noisshiki, Gifu-City, Gifu-Prefecture, 500-8717, Japan; b Department of Gastroenterology, Gifu Prefectural General Medical Center, 4-6-1 Noisshiki, Gifu-City, Gifu-Prefecture, 500-8717, Japan

**Keywords:** CRP, C-reactive protein, CT, computed tomography, OTSC, over-the-scope clip, TTSC, through-the-scope clip, WBC, white blood cell, Spontaneous oesophageal rupture, Endoscopic closure, Over-the-scope clip

## Abstract

**Introduction and importance:**

Spontaneous esophageal rupture is a life-threatening condition caused by a sudden increase in the intraesophageal pressure. While surgery is the mainstay of management for spontaneous esophageal ruptures, in recent years, an increasing number of patients have been managed with endoscopic interventions. We report a case of spontaneous esophageal rupture managed with endoscopic closure using an over-the-scope clip (Ovesco Endoscopy AG, Tübingen, Germany).

**Case presentation:**

A 68-year-old female presented with epigastric pain and left-sided back pain following vomiting. A computed tomography scan revealed mediastinal emphysema and an esophagogram showed leakage from the left side of the lower thoracic esophagus into the mediastinum. The patient was diagnosed with spontaneous esophageal rupture localized to the mediastinum and was treated conservatively. However, she had persistent fever and continuing esophageal leakage on the esophagogram. On the 12th day of admission, a gastrointestinal endoscopy was performed, which found a 10-mm full-thickness longitudinal laceration on the left side of the lower esophagus. Endoscopic closure using an over-the-scope clip was performed. The next day, the patient became afebrile. One week later, esophagogram revealed slight residual leakage and an additional endoscopic closure using an over-the-scope clip was performed; the patient subsequently had an uneventful recovery and was discharged on the 44th day of admission.

**Clinical discussion:**

Endoscopic closure using an over-the-scope clip led to a good outcome in this patient with spontaneous esophageal rupture.

**Conclusion:**

Endoscopic closure using an over-the-scope clip is an effective and minimally invasive technique for selected patients with spontaneous esophageal rupture.

## Highlights

• Spontaneous esophageal rupture is usually treated surgically.
• However, endoscopic interventions can be performed with good outcomes.
• Approach is based on degree of infection in the mediastinum and thoracic cavity.
• We used an over-the-scope clip for esophageal rupture localized to mediastinum.
• The esophageal perforation was closed once the inflammatory response subsided.

## 1 Introduction

Spontaneous esophageal rupture is a longitudinal full-thickness tear of the esophagus due to a sudden increase in intraesophageal pressure. It can be followed by complications, such as mediastinal emphysema, mediastinitis, pneumothorax, empyema, sepsis, and shock, which carry a high risk of mortality. It is reported that the survival rate is substantially decreased unless an accurate diagnosis is made within 24 h and early therapeutic intervention is instituted [1]. Surgery is the mainstay of management for spontaneous esophageal ruptures. However, in recent years, an increasing number of patients with esophageal ruptures have been managed with endoscopic interventions. We report a case of spontaneous esophageal rupture that was managed with endoscopic closure using an over-the-scope clip (OTSC) (Ovesco Endoscopy AG, Tübingen, Germany) and discuss the effectiveness of OTSCs. This work has been reported in line with the Surgical Case Reports guidelines [2].

## 2 Presentation of case

**Fig. 1:**
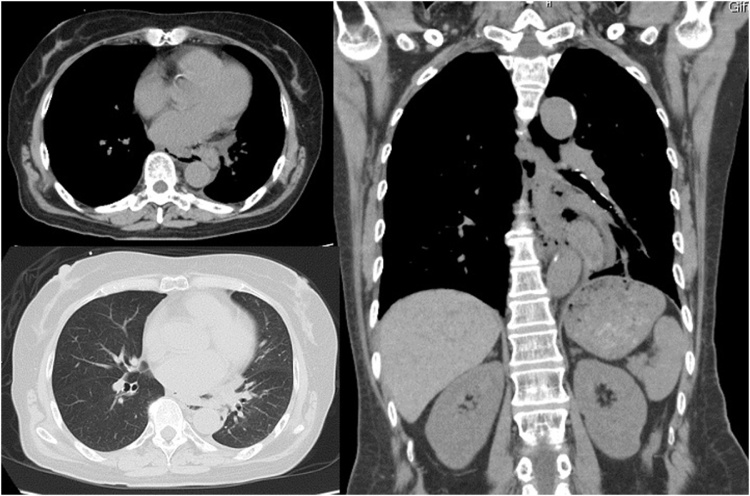
A computed tomography scan showing mediastinal emphysema.

**Fig. 2:**
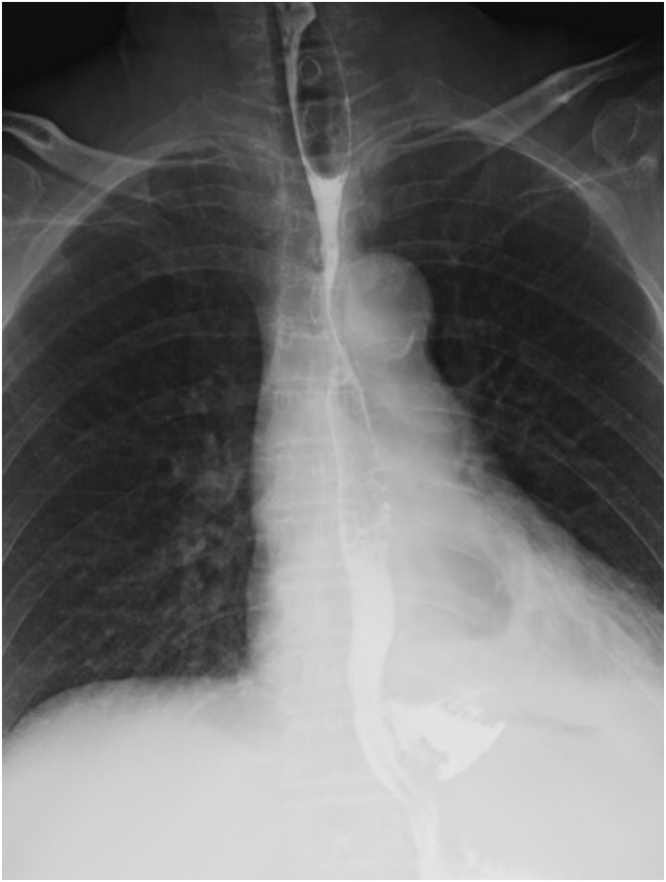
An esophagogram showing leakage from the left side of the lower thoracic esophagus into the mediastinum.

**Fig. 3:**
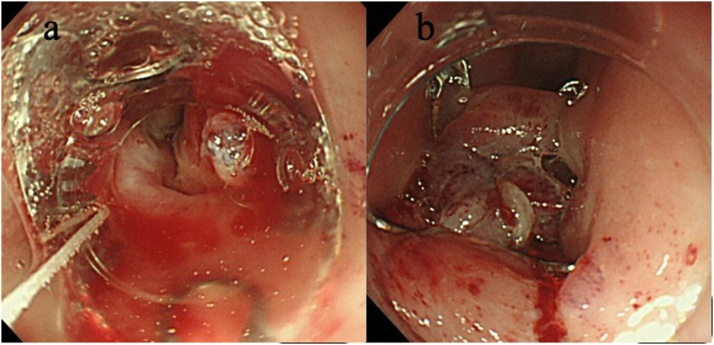
a) Gastrointestinal endoscopy showing a 10 mm full-thickness longitudinal laceration of the left side of the lower esophagus. b) Endoscopic closure using an over-the-scope clip was performed.

**Fig. 4:**
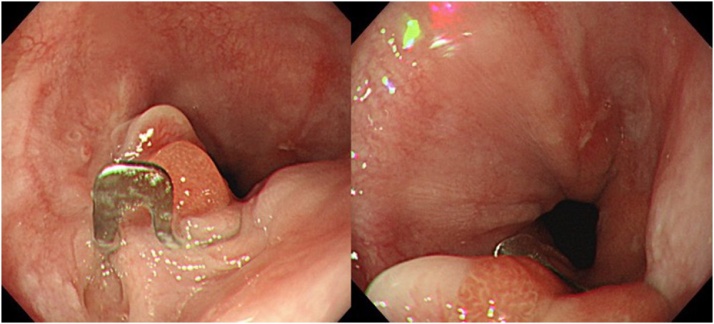
Gastrointestinal endoscopy showing that the previous over-the-scope clips were in-site without any complications, such as stenosis or fistula.

A 68-year-old female presented to the emergency department, using public transport unassisted, with epigastric pain and left-sided back pain following forced vomiting after dinner. There was no relevant past or family history, and she was not using any chronic medication. On arrival, she displayed no obvious signs of illness and looked generally well. She had a blood pressure of 176/90 mm Hg, heart rate of 60 beats/min, temperature of 36.7 °C, respiratory rate of 24 breaths/min, and peripheral oxygen saturation of 96 % in ambient air. There was slight pain in the left upper back during deep breathing. Guarding and rebound tenderness were observed in the upper abdomen. Laboratory data showed a white blood cell (WBC) count of 10,000/μL and C-reactive protein (CRP) of 0.06 mg/dL. Other blood tests were within the normal range. A computed tomography (CT) scan revealed mediastinal emphysema (Fig. 1). An esophagogram showed leakage from the left side of the lower thoracic esophagus into the mediastinum (Fig. 2). A diagnosis of spontaneous esophageal rupture localized to the mediastinum was thus made. Since the patient’s general condition was good and the esophageal rupture was localized to the mediastinum, the patient was initially managed with intravenous broad-spectrum antibiotics, proton pump inhibitors, and total parenteral nutrition. On the day after admission, her general condition did not change; however, laboratory data showed an elevated WBC count of 11,700/μL and CRP of 22.7 mg/dL, and CT showed worsening left pleural effusion. A drainage tube was inserted into the left thoracic cavity, which drained the serous discharge. Over the following days, her symptoms gradually improved. On the 12th day after admission, though a decrease in the WBC count to 3,200/μL and CRP to 5.58 mg/dL was noted, she had a persistent fever and continuing esophageal leakage on the esophagogram. Gastrointestinal endoscopy was performed by the gastroenterologist, which revealed a 10 mm full-thickness longitudinal laceration of the left side of the lower esophagus (Fig. 3a). Thus, endoscopic closure using an OTSC was performed (Fig. 3b). The following day, the patient became afebrile. One week later, an esophagogram revealed slight residual leakage and an additional endoscopic closure using an OTSC was performed. Nine days after endoscopic closure, laboratory data showed a return of WBC counts and CRP to the normal range and oral intake was started. She was discharged on the 32nd day post-endoscopic closure (on the 44th day after admission). Gastrointestinal endoscopy performed 2 months after endoscopic closure showed the previous OTSCs were in-site without any complications such as stenosis or fistula (Fig. 4). Written informed consent was obtained from the patient for the publication of this case report and its accompanying images.

## 3 Discussion

Spontaneous esophageal rupture is classified into two types depending on whether it is associated with pleural rupture or is localized to the mediastinum. The former is associated with a large esophageal perforation and the leakage of air, saliva, and gastric contents into the thoracic cavity, which can lead to pneumonia, empyema, sepsis, and shock. Depending on the extent of the infection, the patient’s general condition may deteriorate and fatality can occur. Meanwhile, spontaneous esophageal rupture localized to the mediastinum is associated with a relatively small perforation and is less likely to cause serious infections.

Spontaneous esophageal rupture without sepsis can be managed conservatively. Cameron et al. have defined the criteria for non-operative treatment as follows: 1) disruption contained in the mediastinum or between the mediastinum and visceral lung pleura, 2) drainage of the cavity back into the esophagus, 3) minimal symptoms, and 4) minimal signs of clinical sepsis [1]. Shaffer et al. have also suggested criteria for non-operative treatment: 1) clinically stable patients, 2) early rupture detection, before major contamination has occurred, and 3) esophageal disruptions that are well contained within the mediastinum or a pleural loculus [3]. It is generally well-accepted that conservative treatment is possible if the patient is in good general condition and has minimal symptoms of infection, especially in cases of spontaneous esophageal rupture localized to the mediastinum.

Treatment consists of drainage of the mediastinum and thoracic cavity, and closure of the esophageal perforation [4]. In patients with spontaneous esophageal rupture with sepsis, early presentation (within 24 h) is commonly managed surgically, but there is no standard strategy for delayed presentation. Traditionally, surgical treatment has predominantly been performed by open thoracotomy. With the spread of endoscopic surgery, thoracoscopic and laparoscopic surgeries are increasingly being performed. In addition, due to advances in endoscopy, successful management has been described using self-expandable, covered metallic stents and clips. Matsuda et al. and Otsuka et al. reported cases of spontaneous esophageal rupture managed with endoscopic clipping [5,6]. In recent years, the OTSC system has been used for the management of gastrointestinal bleeding, fistulas, anastomotic leakage, and perforation, owing to its strong tissue gripping force on all layers of the gastrointestinal wall. Schmidt et al. reported, in a prospective randomized trial of 66 patients at 9 academic referral centers from March 2013 through September 2016, the superiority of OTSC over the through-the-scope-clip (TTSC) in controlling recurrent ulcer bleeding [7]. Gyorgy et al. reviewed the data from 38 articles and 127 patients using a PubMed search and reported that in terms of esophageal perforation closure, TTSC is efficacious in the treatment of less than 10 mm lesions, while larger (<20 mm) lesions can be treated successfully with OTSC [8]. A review of the literature for cases of spontaneous esophageal rupture managed with OTSC successfully revealed three similar cases (Table. 1). Ramhamadany et al. reported the case of a 69-year-old patient with esophageal rupture localized to the mediastinum, in whom OTSC was performed several days after rupture occurrence [9]. Bona et al. reported the case of a 36-year-old patient with pleural rupture, in whom OTSC was performed 10 days after his initial presentation; one week later, a left thoracotomy with pleural decortication was performed for residual left pleural collection [10]. Ali et al. reported the case of a 43-year-old patient with pleural rupture, in whom OTSC was performed 10 days after his initial presentation [11].

**Table 1:** Review of literature of cases of spontaneous esophageal rupture managed with OTSC successfully.

No.	Author	Patient	Type	Cause	location	size	endoscopy intervention (OTSC)	Additonal surgical intervention
1	Ramhamadany	69/Male	localized to the mediastinum	vomiting	lower esophagus	not mentioned	several days after presentation	none
2	Bona	36/Male	pleural rupture	vomiting	left side of esophagogastric junction	10mm	10 days after presentation	left thoracotomy with pleural decortication
3	Ali	43/Male	pleural rupture	vomiting	lower esophagus	15mm	10 days after presentation	none

Compared to surgery, endoscopic interventions using OTSCs are safer, less invasive and easier to perform. However, the degree of infection in the mediastinum and thoracic cavity should be taken into consideration. In patients with pleural rupture, infection control might be achieved by drainage of the thoracic contamination with a chest drainage tube. Although mediastinal contamination is minor and unlikely to cause serious infection in patients with rupture localized to the mediastinum, closure of esophageal perforation may result in inadequate drainage. In iatrogenic esophageal perforation, which results in a low level of contamination due to fasting prior to intervention and rapid diagnosis, good outcomes can be achieved with immediate endoscopic closure. However, in spontaneous esophageal rupture localized to the mediastinum with mediastinal infection, the appropriate timing for closure of perforation is not clear. In our case, the esophageal perforation was closed once the inflammatory response subsided on blood tests. Therefore, good results were obtained. Surgical intervention should also not be debated in cases of deterioration in the patient’s general condition due to abscess formation.

The learning point in this case report is that endoscopic interventions with effective infection control can successfully treat patients with esophageal ruptures.

## 4 Conclusion

Surgery is the mainstay of management for spontaneous esophageal ruptures. However, endoscopic closure with OTSC can be an effective and minimally invasive treatment strategy for selected patients with spontaneous esophageal rupture. Therefore, clinicians should be consider adopting this method to improve patient outcomes.

## Declaration of Competing Interest

The authors report no declarations of interest.

## Sources of funding

This research did not receive any specific grant from funding agencies in the public, commercial, or not-for-profit sectors.

## Ethical approval

This report was reviewed and approved by the Institutional Review Board of Gifu Prefectural General Medical Center.

## Consent

Informed consent was obtained from the patient for publication of this case report.

## Author contribution

Hirokatsu Hayashi: Data Acquisition, Data Interpret and writing of the manuscript.

Narutoshi Nagao & Kenji Yamazaki: management of case.

Ryuichi Asai & Chihiro Tanaka: Supervision, review and editing.

Masahiko Kawai: Supervision, review, editing, and final approval of the version to be submitted.

## Registration of research studies

Not applicable.

## Guarantor

The Guarantor is Hirokatsu Hayashi.

## Provenance and peer review

Not commissioned, externally peer-reviewed.
